# Supplementary material for: An Integrative Computational Approach for Identifying Cotton Host Plant MicroRNAs with Potential to Abate CLCuKoV-Bur Infection
Source: Viruses. 2025 Mar 12;17(3):399. doi: 10.3390/v17030399 (PMC11945813; doi:10.3390/v17030399)
Supplement: Supplementary file 1 [file viruses-17-00399-s001.zip › File S1.pdf]

RNA22 v2 results

```
Resulted have computed and are shown below.  If there are no results shown,
it means your chosen. parameters yielded no results.
Note: The p-value represents the likelihood that the target site loci is
random. That is, a lower p-value represents a greater chance that the loci
contains a valid MRE
```

miR Name	transcript name	leftmost position of predicted target site	folding energy (in -Kcal/mol)	heteroduplex	p value
ghr_miR169a	AM421522.1 Cotton leaf curl Burewala virus _ _India_ Vehari_ 2006_	692	-16.70	GTGTGATGTTTTTCTTGGTTA ||: |:|||||:| GTCCGTTCAGTAGGAACCGAT	1.56E-1
ghr_miR169b	AM421522.1 Cotton leaf curl Burewala virus _ _India_ Vehari_ 2006_	692	-15.40	GTGTGATGTTTTTCTTGGTTA<br>:|: |:|||||:| GGCCGTTTAGTAGGAACCGAC	1.56E-1
ghr_miR390a	AM421522.1 Cotton leaf curl Burewala virus _ _India_ Vehari_ 2006_	1410	-26.80	TGCGCCATTCCCTGCTTGAGCTG |||| ||||| |:|||||| CCGCG-ATAGGGA-GGACTCGAA	9.15E-2
ghr_miR390b	AM421522.1 Cotton leaf curl Burewala virus _ _India_ Vehari_ 2006_	1410	-26.80	TGCGCCATTCCCTGCTTGAGCTG |||| ||||| |:|||||| CCGCG-ATAGGGA-GGACTCGAA	9.15E-2
ghr_miR390c	AM421522.1 Cotton leaf curl Burewala virus _ _India_ Vehari_ 2006_	1410	-26.80	TGCGCCATTCCCTGCTTGAGCTG |||| ||||| |:|||||| CCGCG-ATAGGGA-GGACTCGAA	9.15E-2
ghr_miR169b	AM421522.1 Cotton leaf curl Burewala virus _ _India_ Vehari_ 2006_	1448	-17.70	CTGTGCG-TGAATCCATGGTTG |:| ||: |||| |||:|| GGC-CGTTTAGTAGGAACCGAC	9.15E-2
ghr_miR393	AM421522.1 Cotton leaf curl Burewala virus _ _India_ Vehari_ 2006_	1743	-16.10	GTGTTGGACTGCCAGTCTCTTTGGG<br>:| : ||| :||:||||||:<br>TCTAGTT--ACG-CTAGGGAAACCT	3.19E-1
ghr_miR399e	AM421522.1 Cotton leaf curl Burewala virus _ _India_ Vehari_ 2006_	1747	-17.80	TGGACTGCCAGTCTCTTTGGGCC<br>:|| || |:||||:|| ||| GCC--CCGTTTAGAGGAAACCGT	3.19E-1
ghr_miR399d	AM421522.1 Cotton leaf curl Burewala virus _ _India_ Vehari_ 2006_	1749	-16.30	GACTGCCAGTCTCTTTGGGCC || |:||||:|| ||| GTCCCGTTTAGAGGAAACCGT	3.19E-1
ghr_miR399c	AM421522.1 Cotton leaf curl Burewala virus _ _India_ Vehari_ 2006_	1750	-19.00	ACTGCCAG-TCTCTTTGGGCC ||||: ||||:|| ||| TTCCGGTTGAGAGGAAACCGT	3.19E-1
ghr_miR156c	AM421522.1 Cotton leaf curl Burewala virus _ _India_ Vehari_ 2006_	1751	-17.00	CTGC-CAGTCTCTTTGGGCC ||| || ||||||: |:| CACGAGTGAGAGAAGACTGT	3.19E-1
ghr_miR390a	AM421522.1 Cotton leaf curl Burewala virus _ _India_ Vehari_ 2006_	2443	-15.20	CGTGT-AACTCTCTGCAGAGTTT |:|: |:|||: |||:|| CCGCGATAGGGAGG--ACTCGAA	3.55E-1
ghr_miR390b	AM421522.1 Cotton leaf curl Burewala virus _ _India_ Vehari_ 2006_	2443	-15.20	CGTGT-AACTCTCTGCAGAGTTT |:|: |:|||: |||:|| CCGCGATAGGGAGG--ACTCGAA	3.55E-1

<table>
<tr><th>miR Name</th><th>transcript name</th><th>leftmost position of predicted target site</th><th>folding energy (in -Kcal/mol)</th><th>heteroduplex</th><th>p value</th></tr>
<tr><td>ghr_miR390c</td><td>AM421522.1 Cotton leaf curl Burewala virus _ _India_ Vehari_ 2006_</td><td>2443</td><td>-15.20</td><td>CGTGT-AACTCTCTGCAGAGTTT
 |:|: |:|||: |||:||
CCGCGATAGGGAGG--ACTCGAA</td><td>3.55E-1</td></tr>
<tr><td>ghr_miR7486a</td><td>AM421522.1 Cotton leaf curl Burewala virus _ _India_ Vehari_ 2006_</td><td>2499</td><td>-21.30</td><td>TGAATTTGGG-AAAGTGCTTCCTC
      |||: ||||:|||||||
AGGTGCACCTGTTTCGCGAAGGAA</td><td>7.55E-3</td></tr>
<tr><td>ghr_miR7486b</td><td>AM421522.1 Cotton leaf curl Burewala virus _ _India_ Vehari_ 2006_</td><td>2499</td><td>-21.30</td><td>TGAATTTGGG-AAAGTGCTTCCTC
      |||: ||||:|||||||
AGGTGCACCTGTTTCGCGAAGGAA</td><td>7.55E-3</td></tr>
</table>

<table>
<tr><th>miR Name</th><th>transcript name</th><th>leftmost position of predicted target site</th><th>folding energy (in -Kcal/mol)</th><th>heteroduplex</th><th>p value</th></tr>
<tr><td>ghr_miR7512</td><td>AM421522.1 Cotton leaf curl Burewala virus _ _India_ Vehari_ 2006_</td><td>918</td><td>-16.70</td><td>TGTGTATAACCAGCAGGAAGCT
 :||:||||| :||:| |||
GTACGTATTG-ATGTTCATCGT</td><td>1.88E-1</td></tr>
<tr><td>ghr_miR7491</td><td>AM421522.1 Cotton leaf curl Burewala virus _ _India_ Vehari_ 2006_</td><td>1422</td><td>-15.10</td><td>TGCTTGAGCTGCAGTGATGGGTTCCC
 ||| | |:   :|| :|:|:||
CCGAGTTAGG-AGAGCT-TCTAGGGT</td><td>9.15E-2</td></tr>
</table>

#Please import the downloaded file into Microsoft Excel or other speadsheet software
miRNA_Acc. Target_Acc. Expectation UPE$ miRNA_start miRNA_end
Target_start Target_end miRNA_aligned_fragment alignment
Target_aligned_fragment Inhibition Target_Desc.
Multiplicity
ghr-miR7486a AM421522.1 5.0 -1.0 1 24 2499 2521
AAGGAAGCGCUUUGUCCACGUGGA :. :::. ::::.:::::::
UGAAUUUGGG-AAAGUGCUUCCUC Cleavage Cotton leaf curl Burewala virus - [India: Vehari: 2006] complete genome, clone LA2 1
ghr-miR7486b AM421522.1 5.0 -1.0 1 24 2499 2521
AAGGAAGCGCUUUGUCCACGUGGA :. :::. ::::.:::::::
UGAAUUUGGG-AAAGUGCUUCCUC Cleavage Cotton leaf curl Burewala virus - [India: Vehari: 2006] complete genome, clone LA2 1
ghr-miR2948-5p AM421522.1 5.5 -1.0 1 22 72 93
UGUGGGAGAGUUGGGCAAGAAU ::: .::.:::::::.:
AAGCUUAAAUAAUUCUCCCGCC Cleavage Cotton leaf curl Burewala virus - [India: Vehari: 2006] complete genome, clone LA2 1
ghr-miR7485 AM421522.1 5.5 -1.0 1 24 736 759
AAAGACAUCUUUGAAUUCUUGGAG : .::::: ::...::: :: :::
CCUCAAGAUUUUGGAGAGGUAUUU Cleavage Cotton leaf curl Burewala virus - [India: Vehari: 2006] complete genome, clone LA2 2
ghr-miR7488 AM421522.1 5.5 -1.0 1 21 2558 2578
UUUUGAGUACAGGGGACAAAA ::: : :.::::::. ::: GUUUUUGGCUUGUACUUUAAA
Cleavage Cotton leaf curl Burewala virus - [India: Vehari: 2006] complete genome, clone LA2 1
ghr-miR7492a AM421522.1 5.5 -1.0 1 23 902 924
CUAUAGAACAUGAUCUUUAGCGG :.::: ::.::::: :.::
GAGUUAAUAAUUAUGUUGUGUAU Cleavage Cotton leaf curl Burewala virus - [India: Vehari: 2006] complete genome, clone LA2 1
ghr-miR7492b AM421522.1 5.5 -1.0 1 23 902 924
CUAUAGAACAUGAUCUUUAGCGG :.::: ::.::::: :.::
GAGUUAAUAAUUAUGUUGUGUAU Cleavage Cotton leaf curl Burewala virus - [India: Vehari: 2006] complete genome, clone LA2 1
ghr-miR7492c AM421522.1 5.5 -1.0 1 23 902 924
CUAUAGAACAUGAUCUUUAGCGG :.::: ::.::::: :.::
GAGUUAAUAAUUAUGUUGUGUAU Cleavage Cotton leaf curl Burewala virus - [India: Vehari: 2006] complete genome, clone LA2 1
ghr-miR169b AM421522.1 6.0 -1.0 1 21 2190 2211 CAGCCAAGGAUGAUU-UGCCGG ::::. :: :.:: ::::::: GCGGCGUAAGCGUCGUUGGCUG Cleavage
Cotton leaf curl Burewala virus - [India: Vehari: 2006] complete genome, clone LA21
ghr-miR2949b AM421522.1 6.0 -1.0 1 22 854 875
UCUUUUGAACUGGAUUUGCCGA .:: ::: :. ::::::.::
GUGGACAAUACGCUUCAAAGGA Translation Cotton leaf curl Burewala virus - [India: Vehari: 2006] complete genome, clone LA2 1
ghr-miR2949c AM421522.1 6.0 -1.0 1 22 854 875
UCUUUUGAACUGGAUUUGCCGA .:: ::: :. ::::::.::
GUGGACAAUACGCUUCAAAGGA Translation Cotton leaf curl Burewala virus - [India: Vehari: 2006] complete genome, clone LA2 1
ghr-miR3476-5p AM421522.1 6.0 -1.0 1 21 766 786
UGAACUGGGUUUGUUGGCUGC : .::..:.::::: :. UUUGAUAAUGAGCCCAGUACG
Cleavage Cotton leaf curl Burewala virus - [India: Vehari: 2006] complete genome, clone LA2 1

ghr-miR396a AM421522.1 6.0 -1.0 1 21 1249 1269
UUCCACAGCUUUCUUGAACUG ::: :. .::::::: :: :: CAGAUUCGGAAAGCUAUGAAA
Cleavage Cotton leaf curl Burewala virus - [India: Vehari: 2006] complete genome, clone LA2 1
ghr-miR396b AM421522.1 6.0 -1.0 1 21 1249 1269
UUCCACAGCUUUCUUGAACUG ::: :. .::::::: :: :: CAGAUUCGGAAAGCUAUGAAA
Cleavage Cotton leaf curl Burewala virus - [India: Vehari: 2006] complete genome, clone LA2 1
ghr-miR7493 AM421522.1 6.0 -1.0 1 24 1163 1189 AAUAUUUUAAUAAUU---CAAUCGUCA :::: ::: :.::::: ::::::: UGACAAUUCCUAAGUUAUUUAAAUAUU
Cleavage Cotton leaf curl Burewala virus - [India: Vehari: 2006] complete genome, clone LA2 2
ghr-miR7500 AM421522.1 6.0 -1.0 1 24 675 698
AUCGAGUUAUUCGAGUUAAUCGAG :: : :: :::::::. .:::
GAAGAAUCACACGAAUAGUGUGAU Cleavage Cotton leaf curl Burewala virus - [India: Vehari: 2006] complete genome, clone LA2 1
ghr-miR7503 AM421522.1 6.0 -1.0 1 24 2224 2247
AGAUCGAUGGCUGAACAAGUUAGA :: :: ::::::::::
CUCCUGCUGAUCUUCCAUCGAUCU Translation Cotton leaf curl Burewala virus - [India: Vehari: 2006] complete genome, clone LA2 1
ghr-miR7505 AM421522.1 6.0 -1.0 1 21 2059 2079
UUCAGAAACCAUCCCUUCCUU :.:::. :.::: : ::::: AGGGAGCCGGUGGCUCCUGAA
Cleavage Cotton leaf curl Burewala virus - [India: Vehari: 2006] complete genome, clone LA2 1
ghr-miR7513 AM421522.1 6.0 -1.0 1 21 2551 2571
AAUCAGCCAGGAAUCGUUUGA : :::. .::..:::: .: UGAAAUAGUUUUUGGCUUGUA
Cleavage Cotton leaf curl Burewala virus - [India: Vehari: 2006] complete genome, clone LA2 1
ghr-miR2949a-5p AM421522.1 6.5 -1.0 1 22 320 341
ACUUUUGAACUGGAUUUGCCGA :: . ::. :::.::.::
UUUCCACGCCCGCUUCGAAGGU Translation Cotton leaf curl Burewala virus - [India: Vehari: 2006] complete genome, clone LA2 1
ghr-miR399d AM421522.1 6.5 -1.0 1 21 1749 1769
UGCCAAAGGAGAUUUGCCCUG :: :.::::.:: ::: GACUGCCAGUCUCUUUGGGCC
Cleavage Cotton leaf curl Burewala virus - [India: Vehari: 2006] complete genome, clone LA2 1
ghr-miR399e AM421522.1 6.5 -1.0 1 21 1749 1769
UGCCAAAGGAGAUUUGCCCCG :: :.::::.:: ::: GACUGCCAGUCUCUUUGGGCC
Cleavage Cotton leaf curl Burewala virus - [India: Vehari: 2006] complete genome, clone LA2 1
ghr-miR7485 AM421522.1 6.5 -1.0 1 24 1617 1640
AAAGACAUCUUUGAAUUCUUGGAG :::: ::.:: ::::: :::
GCCCAAUUCUUUAAUGAUGUGUUU Translation Cotton leaf curl Burewala virus - [India: Vehari: 2006] complete genome, clone LA2 2
ghr-miR7491 AM421522.1 6.5 -1.0 1 24 11 34
UGGGAUCUUCGAGAGGAUUGAGCC :: :.::..:.::: .:. ::.:
CGCGCGAUUUUUUCGUGGGCCCUA Cleavage Cotton leaf curl Burewala virus - [India: Vehari: 2006] complete genome, clone LA2 1
ghr-miR7493 AM421522.1 6.5 -1.0 1 24 2095 2118
AAUAUUUUAAUAAUUCAAUCGUCA :: :::::: ::: :.:
UUGCAUUUAAAUUAUGAAAUUGUA Cleavage Cotton leaf curl Burewala virus - [India: Vehari: 2006] complete genome, clone LA2 2
ghr-miR7495a AM421522.1 6.5 -1.0 1 21 539 559
UUACUUUAGAUGUCUCCUUCA :: : :.: :::::::: UUCAGCAUAUAGGUAAAGUAA

Translation Cotton leaf curl Burewala virus - [India: Vehari: 2006] complete genome, clone LA2 1
ghr-miR7495b AM421522.1 6.5 -1.0 1 21 539 559
UUACUUUAGAUGUCUCCUUCA :: : :.: :::::::: UUCAGCAUAUAGGUAAAGUAA
Translation Cotton leaf curl Burewala virus - [India: Vehari: 2006] complete genome, clone LA2 1
ghr-miR7497 AM421522.1 6.5 -1.0 1 23 460 482
ACAUGUGGACUGUCAUAUGGGUU ..:: . :::::: ::::
AGGAUGUACAGAAGUCCAGAUGU Cleavage Cotton leaf curl Burewala virus - [India: Vehari: 2006] complete genome, clone LA2 1
ghr-miR7510a AM421522.1 6.5 -1.0 1 24 703 727 AAGGUC-AUGAUCUUUAGCGGCGUU :.::.:::::.: :::::
UUCUUGGUUAGAGAUCGUAGACCUG Cleavage Cotton leaf curl Burewala virus - [India: Vehari: 2006] complete genome, clone LA2 1
ghr-miR7510b AM421522.1 6.5 -1.0 1 23 806 828
AAGAACAUGAUCUUUAGCGGCGU .: ::.. :::: :::::
AUCGUGAUAGGUAUCAAGUUCUG Cleavage Cotton leaf curl Burewala virus - [India: Vehari: 2006] complete genome, clone LA2 1

```
RNAhybrid
Individual hits
------------------------------------------------------------

dataset: 1
target: AM421522.1
length: 2759
miRNA : ghr-miR156a
length: 20

mfe: -23.5 kcal/mol
p-value: 1.000000e+00

position  2203
target 5'  C   G   G   G     C 3'
            GUU GCU UCU CUGUC
            CGA UGA AGA GACAG
miRNA  3' CA   G   G   A     U 5'

------------------------------------------------------------

dataset: 1
target: AM421522.1
length: 2759
miRNA : ghr-miR156b
length: 20

mfe: -23.5 kcal/mol
p-value: 1.000000e+00

position  2203
target 5'  C   G   G   G     C 3'
            GUU GCU UCU CUGUC
            CGA UGA AGA GACAG
miRNA  3' CA   G   G   A     U 5'

------------------------------------------------------------

dataset: 1
target: AM421522.1
length: 2759
miRNA : ghr-miR156c
length: 20

mfe: -23.5 kcal/mol
p-value: 1.000000e+00

position  2511
target 5' A      C      AG   G  3'
           GUGCUU CUCUUU  UGA
           CACGAG GAGAGA  ACU
miRNA  3'        U      AG   GU 5'

------------------------------------------------------------

dataset: 1
target: AM421522.1
```

length: 2759
miRNA : ghr-miR156d
length: 20

mfe: -23.5 kcal/mol
p-value: 1.000000e+00

```
position  2203
target 5'  C   G   G   G     C 3'
            GUU GCU UCU CUGUC
            CGA UGA AGA GACAG
miRNA  3' CA   G   G   A     U 5'
```

----

dataset: 1
target: AM421522.1
length: 2759
miRNA : ghr-miR160
length: 21

mfe: -25.0 kcal/mol
p-value: 1.000000e+00

```
position  2054
target 5' A     GGGA  CG         G A  3'
           AUAUA    GC  GUGGCUCCU A
           UAUGU    CG  UACCGAGGA U
miRNA  3'       A                G AU 5'
```

----

dataset: 1
target: AM421522.1
length: 2759
miRNA : ghr-miR162a
length: 21

mfe: -23.0 kcal/mol
p-value: 1.000000e+00

```
position  1682
target 5'  A   U    C   AAGA       U 3'
            GGA UGCA AGG    UUGUCGG
            CCU ACGU UCC    AAUAGCU
miRNA  3' GA        C   A            5'
```

----

dataset: 1
target: AM421522.1
length: 2759
miRNA : ghr-miR164
length: 21

mfe: -27.2 kcal/mol
p-value: 1.000000e+00

```
position  1409
target 5' A     CAUU        GAG  G  G 3'
           UGCGC    CCCUGCUU   CU CA
           ACGUG    GGGACGAA   GA GU
miRNA  3'       CAC              G    5'

------------------------------------------------------------

dataset: 1
target: AM421522.1
length: 2759
miRNA : ghr-miR166b
length: 21

mfe: -25.8 kcal/mol
p-value: 1.000000e+00

position  2584
target 5' U             AUUGACUU     AAUUA  G 3'
           GGGG   GGAGCC        UGGUC     GA
           CCCC   CUUCGG        ACCAG     CU
miRNA  3'      UUA                     G        5'

------------------------------------------------------------

dataset: 1
target: AM421522.1
length: 2759
miRNA : ghr-miR167a
length: 21

mfe: -23.8 kcal/mol
p-value: 1.000000e+00

position  1954
target 5' G     UAAGGG  C C     GG  U 3'
           AGGUC      GC G GCAGC  CA
           UCUAG      CG C CGUCG  GU
miRNA  3' A     UA      A       AA    5'

------------------------------------------------------------

dataset: 1
target: AM421522.1
length: 2759
miRNA : ghr-miR167b
length: 21

mfe: -23.8 kcal/mol
p-value: 1.000000e+00

position  1954
target 5' G     UAAGGG  C C     GG  U 3'
           AGGUC      GC G GCAGC  CA
           UCUAG      CG C CGUCG  GU
miRNA  3' A     UA      A       AA    5'
```

----

dataset: 1
target: AM421522.1
length: 2759
miRNA : ghr-miR169a
length: 21

mfe: -28.9 kcal/mol
p-value: 1.000000e+00

```
position  2190
target 5' G   C          G       U 3'
           CGG GUAAG CGUC UUGGCUG
           GUC CGUUC GUAG AACCGAU
miRNA  3'           A    G         5'
```

----

dataset: 1
target: AM421522.1
length: 2759
miRNA : ghr-miR169b
length: 21

mfe: -31.3 kcal/mol
p-value: 1.000000e+00

```
position  2190
target 5' G     UAAG    G       U 3'
           CGGCG    CGUC UUGGCUG
           GCCGU    GUAG AACCGAC
miRNA  3' G     UUA     G         5'
```

----

dataset: 1
target: AM421522.1
length: 2759
miRNA : ghr-miR172
length: 21

mfe: -24.9 kcal/mol
p-value: 1.000000e+00

```
position  2187
target 5' G         AAG     UU  CUG   G 3'
           CUGCGGCGU   CGUCG  GG   UCU
           GACGUCGUA   GUAGU  CC   AGA
miRNA  3'                       UA      5'
```

----

dataset: 1
target: AM421522.1
length: 2759

```
miRNA : ghr-miR390a
length: 21

mfe: -33.4 kcal/mol
p-value: 1.000000e+00

position  1410
target 5' U    CAU     G        G 3'
           GCGC   UCCCU CUUGAGCU
           CGCG   AGGGA GGACUCGA
miRNA  3' C    AU               A 5'

------------------------------------------------------------

dataset: 1
target: AM421522.1
length: 2759
miRNA : ghr-miR390b
length: 21

mfe: -33.4 kcal/mol
p-value: 1.000000e+00

position  1410
target 5' U    CAU     G        G 3'
           GCGC   UCCCU CUUGAGCU
           CGCG   AGGGA GGACUCGA
miRNA  3' C    AU               A 5'

------------------------------------------------------------

dataset: 1
target: AM421522.1
length: 2759
miRNA : ghr-miR390c
length: 21

mfe: -33.4 kcal/mol
p-value: 1.000000e+00

position  1410
target 5' U    CAU     G        G 3'
           GCGC   UCCCU CUUGAGCU
           CGCG   AGGGA GGACUCGA
miRNA  3' C    AU               A 5'

------------------------------------------------------------

dataset: 1
target: AM421522.1
length: 2759
miRNA : ghr-miR393
length: 22

mfe: -22.9 kcal/mol
p-value: 1.000000e+00
```

```
position  1402
target 5' C    AU     CCAU     GC   A  3'
           AGAU  AUGCG    UCCCU  UUG
           UCUA  UACGC    AGGGA  AAC
miRNA  3'      GU     U             CU 5'
```

```
dataset: 1
target: AM421522.1
length: 2759
miRNA : ghr-miR394a
length: 20

mfe: -21.9 kcal/mol
p-value: 1.000000e+00

position  852
target 5'   C        AUAC   U  3'
             GGUGGACA    GCU
             CCACCUGU    CGG
miRNA  3' CCU        CUUA   UU 5'
```

```
dataset: 1
target: AM421522.1
length: 2759
miRNA : ghr-miR394b
length: 20

mfe: -21.9 kcal/mol
p-value: 1.000000e+00

position  852
target 5'   C        AUAC   U  3'
             GGUGGACA    GCU
             CCACCUGU    CGG
miRNA  3' CCU        CUUA   UU 5'
```

```
dataset: 1
target: AM421522.1
length: 2759
miRNA : ghr-miR396a
length: 21

mfe: -22.5 kcal/mol
p-value: 1.000000e+00

position  1225
target 5' C    C            A  U 3'
           CAGU    GGAGGCUGUG GG
           GUCA    CUUUCGACAC CU
miRNA  3'      AGUU             U 5'
```

----

dataset: 1
target: AM421522.1
length: 2759
miRNA : ghr-miR396b
length: 21

mfe: -22.5 kcal/mol
p-value: 1.000000e+00

position 1225

```
target 5' C    C             A  U 3'
           CAGU    GGAGGCUGUG GG
           GUCA    CUUUCGACAC CU
miRNA  3'      AGUU             U 5'
```

----

dataset: 1
target: AM421522.1
length: 2759
miRNA : ghr-miR398
length: 21

mfe: -23.4 kcal/mol
p-value: 1.000000e+00

position 2480

```
target 5'  C A     UG    U   U   3'
            G GGGUG  UUGA GGC
            C CCCAC  GACU UUG
miRNA  3' UU       UG    C   UGU 5'
```

----

dataset: 1
target: AM421522.1
length: 2759
miRNA : ghr-miR399a
length: 21

mfe: -26.3 kcal/mol
p-value: 1.000000e+00

position 2649

```
target 5' A       CAAUUUAUAUU       AA    A 3'
           CUGGAUA           GUCUCCA  UGGC
           GGCCUGU           UAGAGGU  ACCG
miRNA  3'        U                 A     C 5'
```

----

dataset: 1
target: AM421522.1
length: 2759
miRNA : ghr-miR399b

length: 21

mfe: -26.3 kcal/mol
p-value: 1.000000e+00

position 2649
```
target 5' A       CAAUUUAUAUU       AA    A 3'
           CUGGAUA           GUCUCCA  UGGC
           GGCCUGU           UAGAGGU  ACCG
miRNA  3'         U                 A     C 5'
```

----

dataset: 1
target: AM421522.1
length: 2759
miRNA : ghr-miR399c
length: 21

mfe: -24.7 kcal/mol
p-value: 1.000000e+00

position 1752
```
target 5'   U             G   C 3'
             GCCAG UCUCUUU GGC
             CGGUU AGAGGAA CCG
miRNA  3' UUC     G       A   U 5'
```

----

dataset: 1
target: AM421522.1
length: 2759
miRNA : ghr-miR399d
length: 21

mfe: -22.5 kcal/mol
p-value: 1.000000e+00

position 1747
```
target 5'  U  ACU  C         G   C 3'
            GG   GC AGUCUCUUU GGC
            CC   CG UUAGAGGAA CCG
miRNA  3' GU       U         A   U 5'
```

----

dataset: 1
target: AM421522.1
length: 2759
miRNA : ghr-miR399e
length: 21

mfe: -23.9 kcal/mol
p-value: 1.000000e+00

position 1747

```
target 5' U  ACU  C         G   C 3'
           GG   GC AGUCUCUUU GGC
           CC   CG UUAGAGGAA CCG
miRNA  3' G  C    U         A   U 5'
```

----

dataset: 1
target: AM421522.1
length: 2759
miRNA : ghr-miR479
length: 22

mfe: -24.7 kcal/mol
p-value: 1.000000e+00

position 150

```
target 5' A     UUCC    C    U       G 3'
           AUGAG    CCGA ACCG   UCACG
           UACUC    GGCU UGGU   AGUGC
miRNA  3' C                  UAU       5'
```

----

dataset: 1
target: AM421522.1
length: 2759
miRNA : ghr-miR482a
length: 22

mfe: -22.6 kcal/mol
p-value: 1.000000e+00

position 2580

```
target 5' C  U         CCAUUGACUUU  UCAAUU   G 3'
           GU UGGGGGGAG           GG      AGA
           CA ACCCUCCUC           CC      UCU
miRNA  3' C  U         AU           UU         5'
```

----

dataset: 1
target: AM421522.1
length: 2759
miRNA : ghr-miR482b
length: 22

mfe: -27.3 kcal/mol
p-value: 1.000000e+00

position 582

```
target 5' U    C    C  ACCCAUA    U       G 3'
           GGUA UGGG UG       GAGU GGUAAGA
           CCGU ACCC AC       CUCA CCGUUCU
miRNA  3'                         U         5'
```

```
dataset: 1
target: AM421522.1
length: 2759
miRNA : ghr-miR827a
length: 21

mfe: -21.0 kcal/mol
p-value: 1.000000e+00

position  2484
target 5' G  G         CUUGA      G 3'
           GU UGUUGAUGG     AUUUGG
           CA ACAACUACC     UAGAUU
miRNA  3' A  A         AG           5'
```

```
dataset: 1
target: AM421522.1
length: 2759
miRNA : ghr-miR827b
length: 21

mfe: -21.0 kcal/mol
p-value: 1.000000e+00

position  2484
target 5' G  G         CUUGA      G 3'
           GU UGUUGAUGG     AUUUGG
           CA ACAACUACC     UAGAUU
miRNA  3' A  A         AG           5'
```

```
dataset: 1
target: AM421522.1
length: 2759
miRNA : ghr-miR827c
length: 21

mfe: -21.0 kcal/mol
p-value: 1.000000e+00

position  2484
target 5' G  G         CUUGA      G 3'
           GU UGUUGAUGG     AUUUGG
           CA ACAACUACC     UAGAUU
miRNA  3' A  A         AG           5'
```

```
dataset: 1
target: AM421522.1
length: 2759
miRNA : ghr-miR2948-5p
length: 22
```

```
mfe: -26.3 kcal/mol
p-value: 1.000000e+00

position  66
target 5'      C     AAGCUUAAAUA          C 3'
                GCUCA           AUUCUCCCGC
                CGGGU           UGAGAGGGUG
miRNA  3' UAAGAA                          U 5'

------------------------------------------------------------

dataset: 1
target: AM421522.1
length: 2759
miRNA : ghr-miR2949a-3p
length: 21

mfe: -20.2 kcal/mol
p-value: 1.000000e+00

position  2625
target 5' G       ACUCUGGGAAUUGGA        A     3'
           GGCUUUU               GACUGGAU
           UUGAAAA               CUGACCUA
miRNA  3' A                              AACGU 5'

------------------------------------------------------------

dataset: 1
target: AM421522.1
length: 2759
miRNA : ghr-miR2949a-5p
length: 22

mfe: -21.7 kcal/mol
p-value: 1.000000e+00

position  1241
target 5'  A   C       A       A  3'
            GGU  AUCCAG UUCGGAA
            CCG  UAGGUC AAGUUUU
miRNA  3' AG   UU              CA 5'

------------------------------------------------------------

dataset: 1
target: AM421522.1
length: 2759
miRNA : ghr-miR2949b
length: 22

mfe: -21.7 kcal/mol
p-value: 1.000000e+00

position  1241
target 5'  A   C       A       A  3'
```

```
            GGU  AUCCAG UUCGGAA
            CCG  UAGGUC AAGUUUU
miRNA  3' AG   UU              CU 5'
```

```
dataset: 1
target: AM421522.1
length: 2759
miRNA : ghr-miR2949c
length: 22

mfe: -21.7 kcal/mol
p-value: 1.000000e+00

position  1241
target 5'  A   C       A       A  3'
            GGU  AUCCAG UUCGGAA
            CCG  UAGGUC AAGUUUU
miRNA  3' AG   UU              CU 5'
```

```
dataset: 1
target: AM421522.1
length: 2759
miRNA : ghr-miR2950
length: 21

mfe: -31.8 kcal/mol
p-value: 1.000000e+00

position  320
target 5'  U      G   GCUUCGAAG        C 3'
            UUCCAC CCC         GUACGCCG
            AAGGUG GGG         CGUGUGGU
miRNA  3' AU      G   A                  5'
```

```
dataset: 1
target: AM421522.1
length: 2759
miRNA : ghr-miR3476-3p
length: 21

mfe: -22.5 kcal/mol
p-value: 1.000000e+00

position  2481
target 5'  G  GG         A     U 3'
            AG  U  GUGUUG UGGCU
            UC  G  UACAAC ACCGA
miRNA  3' AA  UU AC      A       5'
```

```
dataset: 1
target: AM421522.1
length: 2759
miRNA : ghr-miR3476-5p
length: 21

mfe: -25.8 kcal/mol
p-value: 1.000000e+00

position  422
target 5'  G   G          U AA  G 3'
            GGC AACAGGCCCA G  CA
            UCG UUGUUUGGGU C  GU
miRNA  3' CG   G            AA    5'

------------------------------------------------------------

dataset: 1
target: AM421522.1
length: 2759
miRNA : ghr-miR7484a
length: 24

mfe: -20.1 kcal/mol
p-value: 1.000000e+00

position  1549
target 5' G    GUA        GG  GCUGA      U 3'
           UGCU   CUUUGAUU  AA     GUACAG
           ACGA   GAAACUAG  UU     UAUGUU
miRNA  3' A               A   A          U 5'

------------------------------------------------------------

dataset: 1
target: AM421522.1
length: 2759
miRNA : ghr-miR7484b
length: 24

mfe: -20.1 kcal/mol
p-value: 1.000000e+00

position  1549
target 5' G    GUA        GG  GCUGA      U 3'
           UGCU   CUUUGAUU  AA     GUACAG
           ACGA   GAAACUAG  UU     UAUGUU
miRNA  3' A               A   A          U 5'

------------------------------------------------------------

dataset: 1
target: AM421522.1
length: 2759
miRNA : ghr-miR7485
length: 24
```

```
mfe: -21.8 kcal/mol
p-value: 1.000000e+00

position  473
target 5' G     AU    CUA        G    3'
           UCCAG  GUUC   GAGGAUGU
           AGGUU  UAAG   UUUCUACA
miRNA  3' G     CU               GAAA 5'
```

```
dataset: 1
target: AM421522.1
length: 2759
miRNA : ghr-miR7486a
length: 24

mfe: -30.7 kcal/mol
p-value: 1.000000e+00

position  850
target 5' A            UA      A   3'
           CCG GUGGACAA  CGCUUC
           GGU CACCUGUU  GCGAAG
miRNA  3' A   G        UC      GAA 5'
```

```
dataset: 1
target: AM421522.1
length: 2759
miRNA : ghr-miR7486b
length: 24

mfe: -30.7 kcal/mol
p-value: 1.000000e+00

position  850
target 5' A            UA      A   3'
           CCG GUGGACAA  CGCUUC
           GGU CACCUGUU  GCGAAG
miRNA  3' A   G        UC      GAA 5'
```

```
dataset: 1
target: AM421522.1
length: 2759
miRNA : ghr-miR7487
length: 24

mfe: -23.6 kcal/mol
p-value: 1.000000e+00

position  1575
target 5'   U      G     UC       A 3'
             AC AGU GUCCU  GAGAGUG
```

```
             UG UCA CAGGA  UUCUCAU
miRNA  3' AAU  U         UA       A 5'

------------------------------------------------------------

dataset: 1
target: AM421522.1
length: 2759
miRNA : ghr-miR7488
length: 21

mfe: -24.8 kcal/mol
p-value: 1.000000e+00

position  1443
target 5'     U          G      3'
               UCCCCUGUGC
               AGGGGACAUG
miRNA  3' AAAAC          AGUUUU 5'

------------------------------------------------------------

dataset: 1
target: AM421522.1
length: 2759
miRNA : ghr-miR7489
length: 24

mfe: -23.4 kcal/mol
p-value: 1.000000e+00

position  1451
target 5' U    GAA   A  GUUG      U    3'
           GCGU   UCC UG    UGGCAG
           UGCA   AGG AC    ACCGUU
miRNA  3'     AG       AUA       GUUA 5'

------------------------------------------------------------

dataset: 1
target: AM421522.1
length: 2759
miRNA : ghr-miR7490
length: 24

mfe: -24.1 kcal/mol
p-value: 1.000000e+00

position  563
target 5'  G         UGUUACUCGU   A         G 3'
            UGUUAGUGA          GGU   CUGGGCU
            GCAGUCACU          UCA   GAUCUGA
miRNA  3' UG                     AAA         5'

------------------------------------------------------------

dataset: 1
```

```
target: AM421522.1
length: 2759
miRNA : ghr-miR7491
length: 24

mfe: -27.0 kcal/mol
p-value: 1.000000e+00

position  7
target 5' U   CGCG          U       U 3'
           GGC    CGAUUUUUUCG GGG CCC
           CCG    GUUAGGAGAGC UCU GGG
miRNA  3'     A             U   A   U 5'

------------------------------------------------------------

dataset: 1
target: AM421522.1
length: 2759
miRNA : ghr-miR7492a
length: 23

mfe: -21.3 kcal/mol
p-value: 1.000000e+00

position  708
target 5'  G            A AC    U  3'
            GUUAGAGAUCGU G  CUGU
            CGAUUUCUAGUA C  GAUA
miRNA  3' GG              AA    UC 5'

------------------------------------------------------------

dataset: 1
target: AM421522.1
length: 2759
miRNA : ghr-miR7492b
length: 23

mfe: -21.3 kcal/mol
p-value: 1.000000e+00

position  708
target 5'  G            A AC    U  3'
            GUUAGAGAUCGU G  CUGU
            CGAUUUCUAGUA C  GAUA
miRNA  3' GG              AA    UC 5'

------------------------------------------------------------

dataset: 1
target: AM421522.1
length: 2759
miRNA : ghr-miR7492c
length: 23

mfe: -21.3 kcal/mol
```

p-value: 1.000000e+00

```
position  708
target 5'  G            A AC    U  3'
            GUUAGAGAUCGU G  CUGU
            CGAUUUCUAGUA C  GAUA
miRNA  3' GG              AA    UC 5'
```

----

dataset: 1
target: AM421522.1
length: 2759
miRNA : ghr-miR7493
length: 24

mfe: -22.2 kcal/mol
p-value: 1.000000e+00

```
position  1351
target 5' G         CG  GCUGAUGAUC        G  3'
           GACGGUUGA  UA          UUGAAAUA
           CUGCUAACU  AU          AAUUUUAU
miRNA  3' A         UA                    AA 5'
```

----

dataset: 1
target: AM421522.1
length: 2759
miRNA : ghr-miR7494
length: 23

mfe: -21.6 kcal/mol
p-value: 1.000000e+00

```
position  1299
target 5' G    GAUU       A   UG UCAUG   A  3'
           UUGU    GAACUGG UCC  A     AGU
           AACA    UUUGAUC AGG  U     UCG
miRNA  3'      AU             UG         A  5'
```

----

dataset: 1
target: AM421522.1
length: 2759
miRNA : ghr-miR7495a
length: 21

mfe: -21.4 kcal/mol
p-value: 1.000000e+00

```
position  1596
target 5' A      UU      UUU   G   3'
           UGAAGG  GCAUUU   AAG
           ACUUCC  UGUAGA   UUC
```

```
miRNA  3'        UC      U     AUU 5'
```

----

dataset: 1
target: AM421522.1
length: 2759
miRNA : ghr-miR7495b
length: 21

mfe: -21.4 kcal/mol
p-value: 1.000000e+00

```
position  1596
target 5' A      UU      UUU   G   3'
           UGAAGG  GCAUUU   AAG
           ACUUCC  UGUAGA   UUC
miRNA  3'        UC      U     AUU 5'
```

----

dataset: 1
target: AM421522.1
length: 2759
miRNA : ghr-miR7497
length: 23

mfe: -28.4 kcal/mol
p-value: 1.000000e+00

```
position  363
target 5' C          UG      U     3'
           AGCCCAUAUG  AG CCG
           UUGGGUAUAC  UC GGU
miRNA  3'            UG  A   GUACA 5'
```

----

dataset: 1
target: AM421522.1
length: 2759
miRNA : ghr-miR7498
length: 24

mfe: -30.9 kcal/mol
p-value: 1.000000e+00

```
position  371
target 5' A      CCGUGCU      CCCAU   CCGC       A 3'
           UGUGAG       GCUGCC     UGU    GUCACCA
           ACACUC       UGAUGG     ACA    CAGUGGU
miRNA  3'                     U                  A 5'
```

----

dataset: 1
target: AM421522.1

length: 2759
miRNA : ghr-miR7499
length: 24

mfe: -20.4 kcal/mol
p-value: 1.000000e+00

position 1677

```
target 5' G    A      C  AG         C  3'
           GACC GGAUUG AC  GAAGAUUGU
           UUGG CUUAAU UG  CUUUUAAUA
miRNA  3'                G          UA 5'
```

----

dataset: 1
target: AM421522.1
length: 2759
miRNA : ghr-miR7500
length: 24

mfe: -24.9 kcal/mol
p-value: 1.000000e+00

position 2478

```
target 5' U    GGG  UG             A 3'
           UCGA   UG  UUGA UGGCUUGA
           AGCU   AU  AGCU AUUGAGCU
miRNA  3' G    A    UG    U        A 5'
```

----

dataset: 1
target: AM421522.1
length: 2759
miRNA : ghr-miR7501
length: 24

mfe: -23.7 kcal/mol
p-value: 1.000000e+00

position 2440

```
target 5'    C       AACUCUCUG       U     A 3'
              UUCGUGU         CAGAGUU GAUAU
              AAGCACA         GUCUUAG CUAUA
miRNA  3' AAAA                       U       5'
```

----

dataset: 1
target: AM421522.1
length: 2759
miRNA : ghr-miR7502
length: 24

mfe: -20.0 kcal/mol
p-value: 1.000000e+00

```
position  1656
target 5'   A       AUAA        CG  C 3'
             AUUCAUU    CUGCUGUU  GA
             UAAGUAA    GAUGACAA  UU
miRNA  3' AAG       A           UU  U 5'
```

----

dataset: 1
target: AM421522.1
length: 2759
miRNA : ghr-miR7503
length: 24

mfe: -25.1 kcal/mol
p-value: 1.000000e+00

```
position  1664
target 5'  A     GC       A   G     G 3'
            UAACU  UGUUCGG CCA  GAUU
            AUUGA  ACAAGUC GGU  CUAG
miRNA  3' AG                  AG    A 5'
```

----

dataset: 1
target: AM421522.1
length: 2759
miRNA : ghr-miR7504a
length: 24

mfe: -22.2 kcal/mol
p-value: 1.000000e+00

```
position  131
target 5'   A          C  U   AA   A 3'
             AUGUGGGAUC AC GUU  AUG
             UGCACCUUAG UG CAA  UAU
miRNA  3' UAC             U   AG     5'
```

----

dataset: 1
target: AM421522.1
length: 2759
miRNA : ghr-miR7504b
length: 24

mfe: -22.2 kcal/mol
p-value: 1.000000e+00

```
position  2205
target 5' U    U    U CUG         C 3'
           UGGC  GUC G     UCCUCCU
           ACUG  UAG C     AGGAGGA
miRNA  3' U    UU   U UAAAA        5'
```

```
------------------------------------------------------------

dataset: 1
target: AM421522.1
length: 2759
miRNA : ghr-miR7505
length: 21

mfe: -24.7 kcal/mol
p-value: 1.000000e+00

position  2046
target 5' A     GAAAUAU     GCC   GGCUC     A 3'
           AAGGA       AGGGA   GGU     CUGAA
           UUCCU       UCCCU   CCA     GACUU
miRNA  3'                   A     AA          5'

------------------------------------------------------------

dataset: 1
target: AM421522.1
length: 2759
miRNA : ghr-miR7506
length: 24

mfe: -31.8 kcal/mol
p-value: 1.000000e+00

position  1804
target 5'  G          UCAAUGACGU   A        C 3'
            GUCGACGUCA          UGU CCAGGCGU
            CGGUUGCGGU          ACA GGUCUGUA
miRNA  3' CA                       G          5'

------------------------------------------------------------

dataset: 1
target: AM421522.1
length: 2759
miRNA : ghr-miR7507
length: 24

mfe: -27.5 kcal/mol
p-value: 1.000000e+00

position  2152
target 5' C     GA       GAC         G 3'
           UCUAA  GCCU CU   UUACUGCCU
           GGGUU  CGGA GA   AGUGAUGGA
miRNA  3'       AA    U              A 5'

------------------------------------------------------------

dataset: 1
target: AM421522.1
length: 2759
```

```
miRNA : ghr-miR7508
length: 21

mfe: -22.4 kcal/mol
p-value: 1.000000e+00

position  84
target 5' U       CCUAUUAUAAGU     GUUG  A  3'
           UCUCCCG            ACUUC    CU
           AGAGGGC            UGAAG    GA
miRNA  3' G                        AAAA  AC 5'

------------------------------------------------------------

dataset: 1
target: AM421522.1
length: 2759
miRNA : ghr-miR7509
length: 24

mfe: -21.0 kcal/mol
p-value: 1.000000e+00

position  2172
target 5'  C  C      UU       G     3'
            UG CUG CG  AAGUGCU
            AC GAC GU  UUCACGA
miRNA  3' UA      A  UU       AAACU 5'

------------------------------------------------------------

dataset: 1
target: AM421522.1
length: 2759
miRNA : ghr-miR7510a
length: 24

mfe: -29.1 kcal/mol
p-value: 1.000000e+00

position  695
target 5' U      UUUCUUG            A     G 3'
           GAUGUU       GUUAGAGAUCGU GACCU
           UUGCGG       CGAUUUCUAGUA CUGGA
miRNA  3'                                 A 5'

------------------------------------------------------------

dataset: 1
target: AM421522.1
length: 2759
miRNA : ghr-miR7510b
length: 23

mfe: -22.3 kcal/mol
p-value: 1.000000e+00
```

```
position  786
target 5' G   A                  A  3'
           GCG CUG UGAAGA  AUGUUC
           UGC GGC AUUUCU  UACAAG
miRNA  3'         G      AG      AA 5'

------------------------------------------------------------

dataset: 1
target: AM421522.1
length: 2759
miRNA : ghr-miR7511
length: 24

mfe: -22.6 kcal/mol
p-value: 1.000000e+00

position  47
target 5' G     CAAU      ACGCGCU        A  3'
           UCGGC    CAUAUG       CAAAGCUU
           AGUCG    GUGUAC       GUUUUGAA
miRNA  3' G     AU                       GA 5'

------------------------------------------------------------

dataset: 1
target: AM421522.1
length: 2759
miRNA : ghr-miR7512
length: 21

mfe: -23.5 kcal/mol
p-value: 1.000000e+00

position  918
target 5' U         CA     AAGC     A 3'
           GUGUAUAAC  GCAGG    UGGCA
           UACGUAUUG  UGUUC    AUCGU
miRNA  3' G         A                 5'

------------------------------------------------------------

dataset: 1
target: AM421522.1
length: 2759
miRNA : ghr-miR7513
length: 21

mfe: -22.5 kcal/mol
p-value: 1.000000e+00

position  2328
target 5' U        AAAUGUGCUGA         G  3'
           UCGGAUGG           CCUGGUUGG
           AGUUUGCU           GGACCGACU
miRNA  3'          AA                  AA 5'
```

```
------------------------------------------------------------

dataset: 1
target: AM421522.1
length: 2759
miRNA : ghr-miR7514
length: 24

mfe: -21.4 kcal/mol
p-value: 1.000000e+00

position  1830
target 5' C        G     GUA       G 3'
           AGGCG UC UUACU   UACUUUG
           UCUGC AG AGUGA   GUGAAAU
miRNA  3'       U        AUA       A 5'

------------------------------------------------------------
```

```
#------------------------
# Search parameters
# score <= 9
# mfe ratio >= 0.2
#------------------------

target        AM421522.1
miRNA         ghr-miR156a
score         9
mfe_ratio     0.51
start         2202
seed_gap      0
seed_mismatch 2
seed_gu       0
gap           0
mismatch      4
gu            2
miRNA_3'      CACGAGUGAGAGAAGACAGU
aln           ..|o|.o||.|||.|||||.
target_5'     UCGUUGGCUGUCUGCUGUCC
//
target        AM421522.1
miRNA         ghr-miR156b
score         9
mfe_ratio     0.51
start         2202
seed_gap      0
seed_mismatch 2
seed_gu       0
gap           0
mismatch      4
gu            2
miRNA_3'      CACGAGUGAGAGAAGACAGU
aln           ..|o|.o||.|||.|||||.
target_5'     UCGUUGGCUGUCUGCUGUCC
//
target        AM421522.1
miRNA         ghr-miR156d
score         9
mfe_ratio     0.51
start         2202
seed_gap      0
seed_mismatch 2
seed_gu       0
gap           0
mismatch      4
gu            2
miRNA_3'      CACGAGUGAGAGAAGACAGU
aln           ..|o|.o||.|||.|||||.
target_5'     UCGUUGGCUGUCUGCUGUCC
//
target        AM421522.1
miRNA         ghr-miR169a
score         9
mfe_ratio     0.69
start         2191
seed_gap      0
```

```
seed_mismatch 1
seed_gu       1
gap           0
mismatch      5
gu            2
miRNA_3'      GUCCGUUCAGUAGGAACCGAU
aln           |o|...|..|o||.||||||o
target_5'     CGGCGUAAGCGUCGUUGGCUG
//
target        AM421522.1
miRNA         ghr-miR169b
score         8
mfe_ratio     0.64
start         2191
seed_gap      0
seed_mismatch 1
seed_gu       1
gap           0
mismatch      5
gu            0
miRNA_3'      GGCCGUUUAGUAGGAACCGAC
aln           |.|...||.|o||.|||||||
target_5'     CGGCGUAAGCGUCGUUGGCUG
//
target        AM421522.1
miRNA         ghr-miR172
score         8.5
mfe_ratio     0.59
start         1236
seed_gap      0
seed_mismatch 2
seed_gu       0
gap           0
mismatch      3
gu            3
miRNA_3'      GACGUCGUAGUAGUCCUAAGA
aln           |||oo..o|||||..|||||.
target_5'     CUGUGAGGUCAUCCAGAUUCG
//
target        AM421522.1
miRNA         ghr-miR394a
score         9
mfe_ratio     0.43
start         432
seed_gap      0
seed_mismatch 2
seed_gu       0
gap           0
mismatch      5
gu            0
miRNA_3'      CCUCCACCUGUCUUACGGUU
aln           |....||.||||||.|||.|
target_5'     GCCCAUGAACAGAAAGCCCA
//
target        AM421522.1
miRNA         ghr-miR394b
score         9
```

```
mfe_ratio     0.43
start         432
seed_gap      0
seed_mismatch 2
seed_gu       0
gap           0
mismatch      5
gu            0
miRNA_3'      CCUCCACCUGUCUUACGGUU
aln           |....||.||||||.|||.|
target_5'     GCCCAUGAACAGAAAGCCCA
//
target        AM421522.1
miRNA         ghr-miR396a
score         7
mfe_ratio     0.58
start         1249
seed_gap      0
seed_mismatch 2
seed_gu       0
gap           0
mismatch      2
gu            2
miRNA_3'      GUCAAGUUCUUUCGACACCUU
aln           |||.|o.o|||||||.||.||
target_5'     CAGAUUCGGAAAGCUAUGAAA
//
target        AM421522.1
miRNA         ghr-miR396b
score         7
mfe_ratio     0.58
start         1249
seed_gap      0
seed_mismatch 2
seed_gu       0
gap           0
mismatch      2
gu            2
miRNA_3'      GUCAAGUUCUUUCGACACCUU
aln           |||.|o.o|||||||.||.||
target_5'     CAGAUUCGGAAAGCUAUGAAA
//
target        AM421522.1
miRNA         ghr-miR399d
score         8.5
mfe_ratio     0.49
start         1749
seed_gap      0
seed_mismatch 1
seed_gu       1
gap           0
mismatch      5
gu            1
miRNA_3'      GUCCCGUUUAGAGGAAACCGU
aln           .|..||.|o||||o||.|||.
target_5'     GACUGCCAGUCUCUUUGGGCC
//
```

```
target        AM421522.1
miRNA         ghr-miR2949a-3p
score         8.5
mfe_ratio     0.39
start         2079
seed_gap      0
seed_mismatch 2
seed_gu       0
gap           0
mismatch      4
gu            1
miRNA_3'      AUUGAAAACUGACCUAAACGU
aln           .|o.||.|..||.||||||||
target_5'     AAGAUUCUAUCUAGAUUUGCA
//
target        AM421522.1
miRNA         ghr-miR2949b
score         8.5
mfe_ratio     0.46
start         854
seed_gap      0
seed_mismatch 1
seed_gu       2
gap           0
mismatch      4
gu            1
miRNA_3'      AGCCGUUUAGGUCAAGUUUUCU
aln           .o||..|||.|o.||||||o||
target_5'     GUGGACAAUACGCUUCAAAGGA
//
target        AM421522.1
miRNA         ghr-miR2949c
score         8.5
mfe_ratio     0.46
start         854
seed_gap      0
seed_mismatch 1
seed_gu       2
gap           0
mismatch      4
gu            1
miRNA_3'      AGCCGUUUAGGUCAAGUUUUCU
aln           .o||..|||.|o.||||||o||
target_5'     GUGGACAAUACGCUUCAAAGGA
//
target        AM421522.1
miRNA         ghr-miR7488
score         8
mfe_ratio     0.42
start         2558
seed_gap      0
seed_mismatch 1
seed_gu       2
gap           0
mismatch      4
gu            0
miRNA_3'      AAAACAGGGGACAUGAGUUUU
```

```
aln           .|||.|..|o||||||o.|||
target_5'     GUUUUUGGCUUGUACUUUAAA
//
target        AM421522.1
miRNA         ghr-miR7493
score         7.5
mfe_ratio     0.52
start         1163
seed_gap      0
seed_mismatch 1
seed_gu       0
gap           3
mismatch      2
gu            1
miRNA_3'      ACUGCUA---ACUUAAUAAUUUUAUAA
aln           ||||.||...|.|o|||||.|||||||
target_5'     UGACAAUUCCUAAGUUAUUUAAAUAUU
//
```
